# Fano Resonance Mach–Zehnder Modulator Based on a Single Arm Coupled with a Photonic Crystal Nanobeam Cavity for Silicon Photonics

**DOI:** 10.3390/s25103240

**Published:** 2025-05-21

**Authors:** Enze Shi, Guang Chen, Lidan Lu, Yingjie Xu, Jieyu Yang, Lianqing Zhu

**Affiliations:** 1 School of Instrumentation Science and Opto-Electronics Engineering, Beijing Information Science and Technology University, Beijing 100192, China; 2022020312@bistu.edu.cn (E.S.); guangchen@bistu.edu.cn (G.C.); 2023030001@mail.hfut.edu.cn (Y.X.); yangjieyu@bistu.edu.cn (J.Y.); 2 Guangzhou Nansha Intelligent Photonic Sensing Research Institute, Guangzhou 511462, China

**Keywords:** photonic crystal nanobeam cavities, Mach–Zehnder modulator, Fano resonance, silicon photonics

## Abstract

Recently, Fano resonance modulators and photonic crystal nanobeam cavities (PCNCs) have attracted more and more attention due to their superior performance, such as high modulation efficiency and high extinction ratio (ER). In this paper, a silicon Fano resonance Mach–Zehnder modulator (MZM) based on a single arm coupled with a PCNC is theoretically analyzed, designed, and numerically simulated. By optimizing the coupling length, lattice constant, coupling gap, and the number of holes in the mirror/taper region, the ER of our MZM can achieve 34 dB. When the applied voltage of the MZM is biased at 4.3 V and the non-return-to-zero on–off keying (NRZ-OOK) signal at a data rate of 10 Gbit/s is modulated, the sharpest asymmetric resonant peak and the most remarkable Fano line shape can be obtained around a wavelength of 1550.68 nm. Compared with the traditional nanobeam cavities, along with the varying radii, our PCNC design has holes with a fixed radius of 90 nm, which is suitable to be fabricated by a 180 nm passive silicon photonic multi-project wafer (MPW). Therefore, our compacted lab-on-chip, resonance-based silicon photonic MZM that is coupled with a PCNC has the advantages of superior performance and easy fabrication, which provide support for photonic integrated circuit designs and can be beneficial to various silicon photonic application fields, including photonic computing, photonic convolutional neural networks, and optical communications, in the future.

## 1. Introduction

In recent years, rapidly growing optical transmission capacities and photonic computing densities have required electro-optic modulators (EOMs) to have a higher modulation efficiency and lower component loss [1,2,3,4,5,6]. In particular, a modulator on the silicon-on-insulator (SOI) waveguide has attracted considerable attention in terms of its high integration, high modulation efficiency, high heat tolerance, and compatibility with the complementary metal oxide semiconductor (CMOS) processing technique [7,8,9,10]. Although the most ubiquitous modulators have spectral characteristics with symmetric Lorentzian line shapes, they have common disadvantages such as high loss, low response rates, and large footprints as photonic integrated circuits in the application of photonic computing [11,12,13,14]. Therefore, a novel silicon modulator with superior performance is required to solve the above-mentioned bottlenecks.

Compared with MZMs [15,16,17] and micro-ring resonators (MRRs) [18,19,20], the Fano resonance modulator has a high modulation efficiency, high ER, and sharp asymmetric resonance peaks, which mean that its wavelength tuning range is comparatively narrow when the optical intensity is altered from the minimum to maximum. Therefore, a photonic crystal electro-optic MZM is proposed, where the photonic crystal is implemented in regions with distinct positive and negative doping concentrations [21]. Although this component achieves a modulation speed of 10 Gbit/s, the driving voltage of this modulator is as high as (~7 V), also with large footprints. Furthermore, EOM side-coupled with a PCNC-loaded graphene/Al_2_O_3_ multilayer stack (GAMS) is proposed, which is modulated by the features of phase change materials with circular holes in the center of the PCNC [22]. However, this modulator exhibits an indistinct Fano line shape, a low ER of less than 10 dB, and a small hole radius (<90 nm), which pose challenges for passive MPW fabrication. Another reconfigurable silicon Fano resonance modulator with the help of thermal control is demonstrated, whose coupling and transmission coefficients are tunable by covering several phase shifters on the MRR and MZM, respectively [23]. Its footprint is large. And then, a thin-film lithium niobate (TFLN) modulator with an ultra-high modulation efficiency of 1.02 V·cm and a 3 dB electro-optic bandwidth of 108 GHz is proposed [24]. However, its ER (4.7 dB) is relatively lower than that of our designed Fano resonance modulator (34 dB). Moreover, its size (500 µm × 5 mm), which is much larger than that of our design, makes it less suitable for on-chip integration. Therefore, a design for a Fano resonance MZM with a small footprint, high ER, and easy fabrication is needed.

In this paper, a comprehensive scheme is proposed by utilizing PCNC holes with an equal radius of 90 nm and tuning the lattice constant of a PCNC to create equivalent discrete states. There are interferences between the discrete states from one arm of the MZM coupled with the PCNC and the continuous states from the other arm of the MZM at a multi-mode interferometer (MMI), respectively, which excites the Fano resonance at a wavelength of 1550.68 nm. Here, the ER for our designed Fano modulator is nearly 34 dB. As the holes of the PCNC are large enough (~90 nm), our component can be easily fabricated by utilizing a mature 180 nm microelectronic foundry that is suitable for a standard full-process silicon photonic passive MPW. Obviously, the processing cost and operation difficulty of our component structure are notably reduced. Above all, our proposed scheme of a Fano resonance MZM coupled with a PCNC has apparent significance for the design, fabrication, and development of a high-performance EOM and provides a reasonable solution for potential applications in the fields of photonic on-chip computing, photonic integrated circuits, optical storage, etc., in future.

## 2. Model and Theoretical Analysis

The asymmetric MZM is designed to have an optical phase difference in the two arms. A reverse bias voltage is applied to the P–N junction for the carrier-depletion MZM, which causes changes in the carrier concentration. Depending on the free-carrier dispersion effect, the change in the refractive index of the waveguide material will affect the effective refractive index of the optical mode, introducing phase changes in the optical signals in both arms. Intensity modulation is obtained by interferences between the two outputs of the MMI. Here, the corresponding output optical signals and the optical phase differences of the two arms are expressed as follows [25,26]:

(1) $$E_{1}=\frac{E_{\mathrm{in}}}{2}e^{-j\left(\omega_{0}t+\varphi_{1}\right)}e^{-\alpha L_{1}}$$

(2) $$E_{2}=\frac{E_{\mathrm{in}}}{2}e^{-j\left(\omega_{0}t+\varphi_{2}\right)}e^{-\beta L_{2}}\times T(\omega )$$

(3) $$\Delta \varphi =\frac{2\pi }{\lambda }\times \Delta n_{\mathrm{eff}}\times \Delta L.$$

where the loss coefficients of the upper and the lower arms are *α* and *β*, respectively, and Δ*n_eff_* expresses the difference between the effective refractive indices of the two arms with the fundamental modes. *λ* and *ω*_0_ indicates the resonant wavelength and the angular frequency of the MZM output optical signal, respectively, and Δ*L* = *L*_2_ − *L*_1_ is the difference between the lengths of the upper and the lower arms. In addition, *φ_rf_* = π*V*/*V_π_* represents the phase change introduced by the loaded radio frequency signal on the electrodes, and the phase difference is written as Δ*φ* = *φ*_2_ − *φ*_1_, arising from the change in refractive indices due to the loaded electrical signals on the upper and the lower arms, respectively. Also, the transfer function of the phase response is noted as the *T*(*ω*) generated by the PCNC structure. Therefore, the total electrical field intensity of the output optical signal for the MZM is expressed as follows:

(4) $$E_{\mathrm{out}}=\frac{E_{\mathrm{in}}}{2}\left[e^{-j\left(\omega_{0}t+\varphi_{1}\right)}e^{-\alpha L_{1}}+e^{-j\left(\omega_{0}t+\varphi_{2}\right)}e^{-\beta L_{2}}\times T(\omega )\right].$$

**Figure 1:**
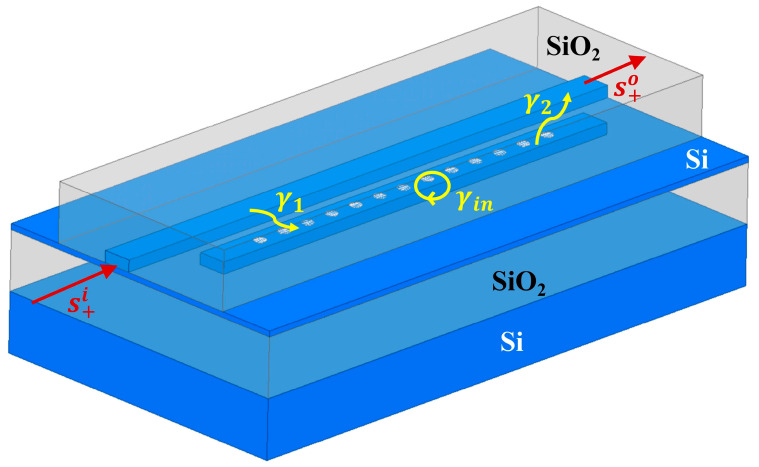
The structural diagram of the side-coupled photonic crystal nanobeam cavity.

As shown in Figure 1, the silicon PCNC and the bus waveguides are etched on the SOI platform, with an etching depth of 220 nm. The optical field propagation process in the structure of a single arm coupled with a PCNC is also illustrated. The optical wave first enters the left side of the bus waveguide, and then its corresponding resonant component is coupled into the nanobeam cavity and later reversely coupled from the cavity to the bus waveguide. Finally, the total optical signal goes out from the right port of the bus waveguide. Thus, according to the temporal coupled-mode theory [27], the transfer function *T*(*ω*) of the coupling structure is expressed as follows [28]:

(5) $$T(\omega )={\left|\frac{s_{+}^{o}\left(t\right)}{s_{+}^{i}\left(t\right)}\right|}^{2}={\left|-j+\frac{2\sqrt{\gamma_{1}\gamma_{2}}}{\gamma_{1}+\gamma_{2}+\gamma_{\mathrm{in}}+j\left(\omega_{o}-\omega \right)}\right|}^{2},$$

where *γ*_1_ and *γ*_2_ are the decay rates from the bus waveguide to the nanobeam cavity and from the nanobeam cavity to the bus waveguide, respectively. The resonant frequency of the nanobeam cavity is *ω*_0_. *γ*_in_ represents the intrinsic loss of the cavity, which is the decay rate of the cavity energy due to non-coupling mechanisms such as absorption and scattering. It is related to the intrinsic quality factor (*Q_in_*) of the cavity by the formula *γ*_in_ = *ω*_0_/(2*Q_in_*), where *ω*_0_ is the resonance frequency of the cavity. *γ*_1_ and *γ*_2_ represent the decay rates of the cavity energy coupled into the cavity from the bus waveguide and coupled out of the cavity back into the bus waveguide, respectively. A larger *γ*_1_ or *γ*_2_ indicates a stronger coupling between the cavity and the waveguide, making it easier for energy to couple from the cavity to the waveguide. Obviously, while fabricating the PCNC, the coupling gap between the bus waveguide and the nanobeam cavity can remarkably affect the coupling decay rates, which directly causes the coupling loss to the PCNC. Therefore, if the fabrication tolerance is limited, slight manufacturing deviations can lead to fluctuations in the coupling coefficient, thereby hindering the component performance. According to the relationship between the output power and the amplitude of the optical signal $P=E_{\mathrm{out}}\cdot E_{\mathrm{out}}^{*}$, the optical power of the output signal can be represented as follows:

(6) $$P=\frac{{E_{\mathrm{in}}}^{2}}{4}[e^{-2\alpha L_{1}}+e^{-2\beta L_{2}}T^{2}(\omega )+2e^{-\alpha L_{1}}e^{-\beta L_{2}}T(\omega )\cos(∆\varphi -\varphi_{t})],$$

where the *φ_t_* represents the phase change induced by *T*(*ω*). The central wavelength of the resonant component for the output optical wave is around 1550 nm in the C band. The optical path difference between the two arms is 10 µm. The free spectral range (*FSR*) is expressed in terms of wavelength (m) [29], as follows:

(7) $$\mathrm{FSR}\left[m\right]=\frac{\lambda^{2}}{n_{g}\times ∆L},$$

where *c*_0_ is light speed in vacuum, and *n_g_* is the group refractive index of the waveguide that has the following expression:

(8) $$n_{g}=n_{\mathrm{eff}}\left(\lambda \right)-\lambda \times \frac{dn_{\mathrm{eff}}}{d\lambda }.$$

Meanwhile, to obtain velocity matching between the light and traveling wave electrodes, the relation between the effective refractive index *n_eff_*(*λ*) and the wavelength is numerically simulated, and the slope rate at around 1550 nm is theoretically calculated. As there is a wavelength drift for the FSR corresponding to the adjustment of a DC bias voltage, the phase change component Δ*φ* introduced by the plasma dispersion effect can be calculated as follows:

(9) $$∆\varphi =\frac{2\pi ∆\lambda }{\mathrm{FSR}},$$

where Δ*λ* represents the wavelength shift of the FSR. Therefore, the modulation efficiency of the EOM can be obtained as *V_π_L*, where *V_π_* is the required driving voltage corresponding to a phase shift of π radians with the optimal waveguide length of *L*.

## 3. Component Structural Design

**Figure 2:**
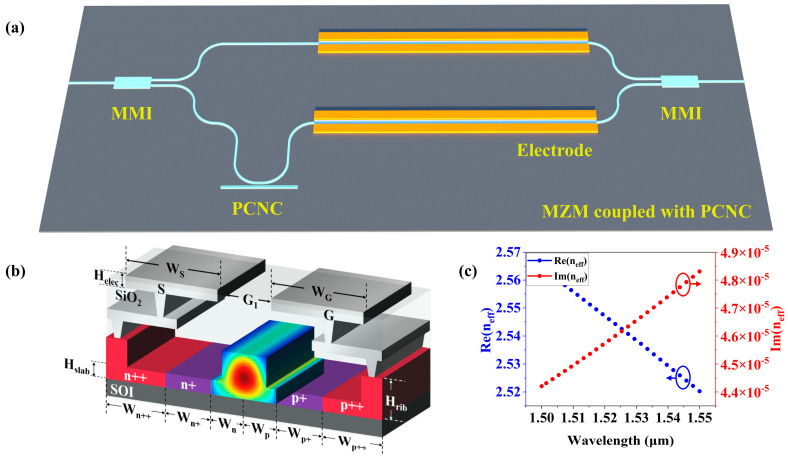
Structure of MZM with a single arm coupled with a PCNC. (**a**) Schematic diagram of MZM with PCNC. (**b**) Cross-sectional view of MZM and optical waveguide mode. (**c**) Variations in effective refractive index with wavelength.

As illustrated in Figure 2a, the overall structure of the Fano resonance MZM design consists of an asymmetric MZM and a PCNC that is coupled to one arm of the MZM. In Figure 2b, the cross-sectional diagram of the modulator doping region is based on the SOI waveguide. The resistivity of the silicon substrate layer is 750 ohm·cm with the thickness of 300 µm, and the thickness of the SiO_2_ insulating layer is 2 µm, which is used to separate the silicon waveguide layer from the substrate layer. A 220 nm thick silicon waveguide layer covers the SiO_2_ layers with a ridge waveguide. Aiming to guarantee the conditions of a single-mode transmission at a wavelength of around 1550 nm in the C band by numerical simulation, the structural parameters such as the ridge waveguide width *W*, the flat layer height *H_slab_*, and the ridge height *H_rib_* are chosen as 450 nm, 90 nm, 130 nm, respectively, which maintain the optical wave propagation direction and the low propagation loss. As shown in Figure 2b, the distribution of the optical field modes shows that the optical signal energy is remarkably confined in the ridge waveguide. Additionally, as depicted in Figure 2c, the relationship between the effective refractive index and the wavelength demonstrates that the effective refractive index at a wavelength of 1550 nm is around 2.52. Moreover, the length of the upper and the lower arm are 1.01 mm and 1.02 mm, respectively.

As shown in Figure 2b, the metal electrode chosen for the design is aluminum, which adopts the configuration of the co-planar metal strips. With the distance between the electrodes (G_1_) and the thickness of the electrode (H_elec_), the widths of the electrodes G and S are denoted as W_G_ and W_S_, respectively. Through electromagnetic simulation, a set of electrode parameters with the impedance of 50 ohm are obtained. Ion implantation is used for doping in the carrier-depletion MZM. The three-dimensional finite-difference time-domain (3D FDTD) method and Ansys Lumerical CHARGE (2022 R2) are employed to achieve a high modulation efficiency of the waveguide. Moreover, CHARGE is employed to calculate the carrier distribution, and FDTD is used to compute the transmission spectrum in the waveguide. According to the different doping concentrations, the heavily doped regions (p++/n++), the lightly doped regions (p+/n+), and the P–N junction are formed, respectively. It is worth noting that the holes have a lower absorption than the electrons, i.e., a higher concentration of the holes than that of the electrons, which is beneficial to a larger phase change [30,31]. The heavily doped region connects the electrodes to the waveguide in order to obtain better ohmic contact. In addition, the lightly doped region connects the heavily doped region with the P–N junction, which affects the modulation efficiency. The reasonable design of the P–N junction can improve the modulation efficiency, the insertion loss, and the capacitance. All these parameters are organized in Table 1. Finally, the data are integrated into INTERCONNECT to perform the numerical calculations for the MZM. Under this set of parameters, the modulator exhibits a modulation efficiency of 1.6 V·cm. And the on-chip insertion loss includes a propagation loss from the doping-induced waveguide (2.3 dB/mm) and a coupling loss from the MMI (each 0.5 dB). Thus, the total on-chip insertion loss is 3.3 dB (2.3 dB/mm × 1 mm + 0.5 dB × 2 = 3.3 dB). Furthermore, when a bias voltage of 4.3 V is applied, the DC capacitance is approximately equal to 0.244 fF/µm.

**Figure 3:**
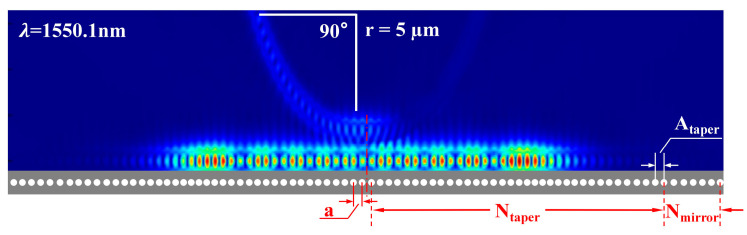
Optical waveguide mode of PCNC. *a*: lattice constant, *N_taper_*: the number of holes in the taper region, *N_mirror_*: the number of holes in the mirror region, *A_taper_*: the maximum lattice constant of holes in the taper region.

As illustrated in Figure 3, the structure of the two-dimensional PCNC is centrosymmetric, which consists of two tapers in the middle region and two mirrors at the left and right sides of the nanobeam cavity, respectively. The red part represents the strongest optical intensity distribution in the PCNC waveguide, and the hole radii of both tapers and mirrors of the PCNC are selected as 90 nm. In our PCNC, the duty cycle of a lattice can be expressed as *f* = π*r*^2^/(*ac*), where *a* is the lattice constant, and *c* represents the waveguide width of the nanobeam cavity. In the taper region, the number of the asymptotic holes *N_taper_* is 40, and the distance between the adjacent holes increases gradually from the center to both sides, which varies according to the following formula: *a*_i_ = *a* − (*i* − 1)^2^(a − *A_taper_*)/(*N_taper_* − 1)^2^, (*i* increases from 1 to *N_taper_*) [32]. The lattice constant *a* of the hole in the center is 0.3 µm, and the maximum lattice constant of the asymptotic region *A_taper_* is 0.43 µm, which makes the light obtain a Gaussian mirror image at the center of the nanobeam cavity. The number of the mirror holes *N_mirror_* is five, and the lattice constant of the mirror holes *A_mirror_* is equal to *A_taper_*, which allows for the formation of an efficient reflective region to enhance the light confinement, as well as improves the light transmission to obtain a high quality-factor (Q-factor). In addition, the coupling gap between the bus waveguide and the PCNC is 0.18 µm. The approach of introducing the discrete states by changing the lattice constant while using holes with the same radius (90 nm), rather than gradually varying radii or using radii smaller than 90 nm, is notable. Also, it was noted that the discrete states, the intrinsic properties created by the PCNC, can interfere with the continuous state of the other arm of the MZM, which generates a Fano resonance. The more superior the performance of the discrete states in the PCNC, the higher the ER is.

**Figure 4:**
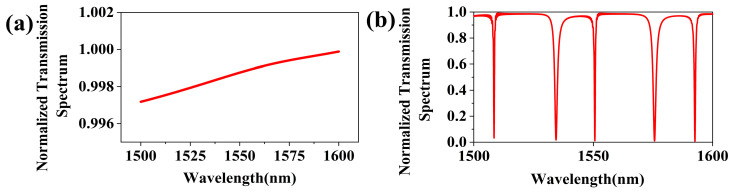
Normalized transmission spectra of (**a**) straight waveguide, and (**b**) two-dimensional PCNC.

Furthermore, the performance of the PCNC is investigated by numerical simulations. A comparison of the normalized transmission spectra between the straight waveguide and the PCNC is shown in Figure 4a,b. The width of the straight waveguide is 0.45 µm, and the width of the PCNC and the bus waveguide are both 0.45 µm. Additionally, the coupling length *L_coupling_* between the PCNC and the bus waveguide is 1 µm, along with *a* = 0.3 µm, *A_taper_* = 0.43 µm, *gap* = 0.18 µm, *N_taper_* = 40, and *N_mirror_* = 5. In Figure 4b, it can be seen that there are discrete states produced in the nanobeam cavity, and the resonant wavelength such as 1550.68 nm is coupled into the nanobeam cavity.

## 4. Numerical Simulation and Results Discussion

**Figure 5:**
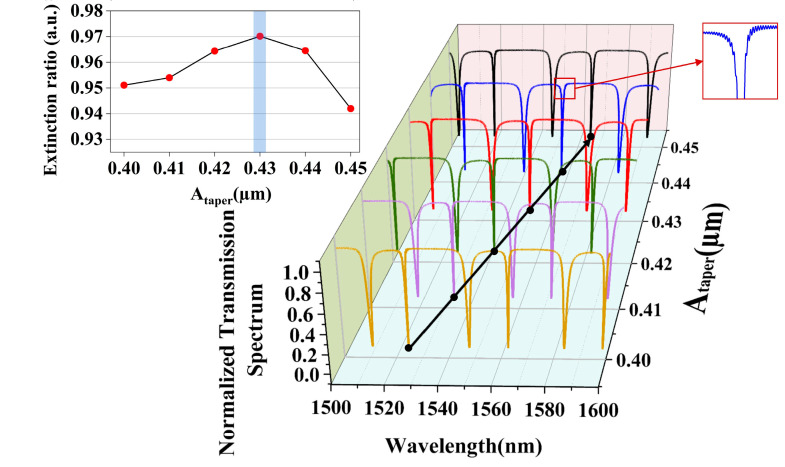
Variations of transmission spectra and ER for PCNC with *A_taper_*.

In this section, the influence of the structural parameters on the performance of the PCNC is numerically simulated after the component design. In order to achieve a high Q-factor, the FDTD model is utilized to analyze the optimal values of *A_taper_*, *N_taper_*, *N_mirror_*, and gap. Initially, the PCNC is engineered on a standard SOI substrate. The single-mode light enters from the upper left port of the bus waveguide and exits from the upper right port. Upon completion of the structural setup, keeping the other parameters constant (*gap* = 0.18 μm, *N_taper_* = 40, and *N_mirror_* = 5), the value of *A_taper_* is gradually varied from 0.40 μm to 0.44 μm. The resulting variations in the normalized transmission spectra and the corresponding ER obtained by numerical simulation are presented in Figure 5 and its inset, respectively. The spectra illustrated by the different colored lines represent different *A_taper_*. It can be seen that with the increase in *A_taper_*, the spectra are gradually red-shifted, and the black line in Figure 5 illustrates this wavelength shift trend. At the same time, the spectral position of a resonant wavelength of 1550.68 nm can be obtained with *A_taper_* = 0.43 µm. Furthermore, the length of the taper region also increases along with the increase in *A_taper_*. 

Meanwhile, with the increase in *A_taper_*, the reason why the ER fluctuates at the resonant wavelength marked with a black line in Figure 5 and the transmission spectra curve at the resonant wavelength becomes gradually asymmetric is that the mirror strength at the different lattice constant changes the reflective optical intensity of the PCNC. As the length of the mirror region increases, the light intensity oscillating within the nanobeam cavity becomes too strong, resulting in mutual interference and causing the spectrum to jitter.

**Figure 6:**
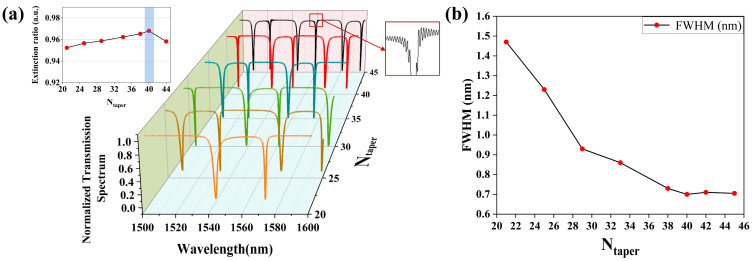
(**a**) Variations in transmission spectra and ER for PCNC with *N_taper_*. (**b**) Relationship between FWHM and *N_taper_*.

Following this, the impact of the number of holes in the taper region on the nanobeam cavity is investigated. Here, the value of *N_taper_* is selected from 21 to 45. As shown in Figure 6a, the variations in the normalized transmission spectra as illustrated by the different colored lines represent the relationships with different *N_taper_*, and the corresponding ERs are demonstrated by the respective calculations. It can be seen from Figure 6a that with the increase in *N_taper_* (21 ≤ *N_taper_* ≤ 45), the spectral width of the resonant peak grows narrower, and the intervals between the adjacent resonant peaks also decrease, while the confinement of the nanobeam cavity becomes stronger, such that the full width at half maximum (FWHM) of the PCNC gradually improves with the same trend. Here, the Q-factor is defined as *ω*_0_/FWHM, and the variation of the FWHM with *N_taper_* is shown in Figure 6b. It is seen from Figure 6b that the performance of the FWHM gradually improves with the increase in *N_taper_*. However, when *N_taper_* > 40, the spectra around the resonant wavelength start to fluctuate dramatically, which leads to undesired reflections of the cavity, making the spectra asymmetric and lowering the confinement of the optical field. The amount of holes in the taper region can not only lead to excessive scattering during the propagation of light, thereby increasing the optical loss, but also cause a mismatch between the modes in the taper region and the entire nanocavity thus lowering the light coupling efficiency. Thus, this effect could affect the resonant characteristics and confinement capabilities of the PCNC corresponding to the light propagation. After the theoretical analysis and numerical simulation, the optimized value of *N_taper_* is chosen as 40. Therefore, it can be seen that a reasonable selection of *N_taper_* matters to a considerable extent, which can bring about a high Q-factor and a high ER to the PCNC and determine the performance of a component or even the entire designed system. 

**Figure 7:**
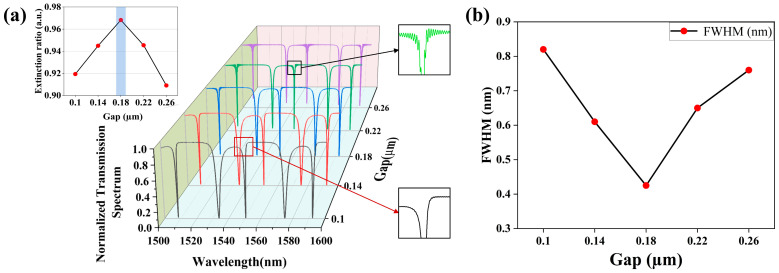
(**a**) Variations in transmission spectra and ER for PCNC with Gap. (**b**) Relation between FWHM and Gap.

Furthermore, the coupling gap between the nanobeam cavity and the bus waveguide can significantly affect the component performance, which is initially iterated from 0.1 µm to 0.26 µm. As shown in Figure 7a, the variations in the normalized transmission spectra as illustrated by the different colored lines represent the relationships with different coupling gaps, and the corresponding ERs are demonstrated by their respective calculations. Obviously, the coupling effects and the spatial distribution of the optical energy can be remarkably affected by the coupling gap between the bus waveguide and the cavity. Also, variations in the FWHM with the different gaps are shown in Figure 7b, where the performance of the FWHM initially improves and then deteriorates after the gap of 0.18 μm. When the coupling gap increases from a distance of zero, the optical signal will sequentially experience the over-coupled (gap < 0.1 μm), critically coupled (0.1 μm < gap < 0.22 μm), and under-coupled (gap ≥ 0.22 μm) states, which can determine the optical waveguide mode coupled from the bus waveguide to the cavity at a specific resonant wavelength. When the gap is ultra narrow, the discrete states do not appear in the spectrum due to the over-coupled state. With the increase in the gap, the discrete states gradually occur. Yet, the spectrum near the discrete states possesses asymmetry, with very low optical power on one side. When the gap is between 0.1 μm and 0.22 μm, the critically coupled state is generated, and the discrete states in the spectrum show a superior performance in optical intensity confinement. However, when the gap exceeds 0.22 μm, the discrete states gradually disappear. When critical coupling is achieved, the optical wave transiting the bus waveguide is maximally coupled to the PCNC if the input signal oscillates at its resonant wavelength. Therefore, after simulation verification and optimization, the optimal performance such as the minimum coupling loss, Q-factor = 2.247 × 10^3^, and ER = 0.97 for the PCNC component can be obtained at a resonant wavelength of 1550.68 nm, when gap = 0.18 µm and *L_coupling_* = 1 µm. Thanks to our coupling gap not being ultra narrow, extra fabrication difficulties are avoided.

**Figure 8:**
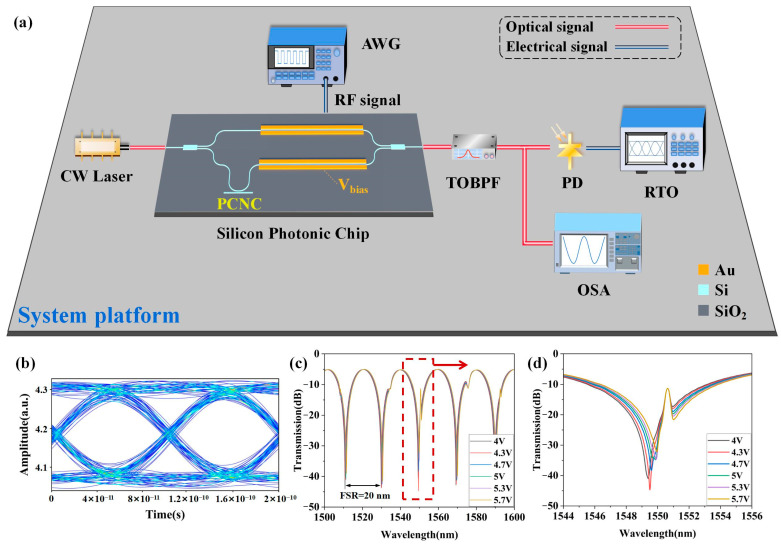
System platform and performance for designed MZM with a single arm coupled with a PCNC. (**a**) Schematic diagram of system platform. (**b**) Eye diagram of NRZ-OOK signal at 10 Gbit/s. (**c**) Transmission spectra of Fano resonance modulator. (**d**) Enlarged spectrum of Fano line shape in detail.

Overall, as illustrated in the schematic diagram of Figure 8a, a comprehensive platform consisting of a silicon photonic chip and discrete components is numerically simulated in Ansys Lumerical INTERCONNECT (2022 R2) to demonstrate the system performance. Firstly, the system link is constructed in the simulation software, as illustrated in Figure 8a. The performance of the PCNC, which is of importance to the system operation, can be directly characterized using the Optical N-port S-parameter element (SPAR) in Ansys Lumerical INTERCONNECT. Also, the S-parameters of the PCNC are imported into this element, and then the SPAR is connected to one arm of the MZM using a waveguide. The optical signal with a power of 10 mW and a central wavelength of 1550.68 nm from a CW laser transmits into the silicon photonic chip, which is our MZM design based on a single arm coupled with a PCNC, and the NRZ-OOK signal *V_t_* at the bit rate of 10 Gbit/s generated from an arbitrary waveform generator (AWG) and the bias voltage *V_bias_* are connected to the electrical input ports of the MZM, respectively. Here, the output optical signal from the silicon chip design is finely filtered by a tunable optical band pass filter (TOBPF) that is used to reduce the out-of-band noise, as well as improve the out-of-band rejection ratio and the optical signal to noise ratio (OSNR). The central wavelength of the TOBPF is set to 1550.68 nm, with a filtering bandwidth of 0.8 nm. The spectrum of this optical signal after the TOBPF is observed by an optical spectrum analyzer (OSA) to analyze and adjust the performance of this optical filtering.

Then, the signal received is observed by some signal analyzers. An ideal PIN photodetector is configured with a responsivity of 1 A/W and set to operate at a wavelength of 1550.68 nm, which is employed to convert the optical signal to the output photocurrent. And the eye diagram of our final output electrical signal can be observed and analyzed by a real-time oscilloscope (RTO), as shown in Figure 8b, where a clear profile with enough openness of an eye diagram is obtained. Herein, the brightest color in the eye diagram expresses the strongest intensity of the overlapped signal parts. When the modulation voltage of the MZM ranges from 4 V to 5.7 V, the transmission spectra from 1500 nm to 1600 nm with the same FSR are as illustrated in Figure 8c. Moreover, as depicted in Figure 8d, the spectral components from 1544 nm to 1556 nm are highlighted, which enlarges the Fano line shape of Figure 8c in detail. Thus, for the spectra of the Fano resonance MZM coupled with a nanobeam cavity within a single FSR around 1550 nm, when the applied voltage is biased at 4.3 V, i.e., the optimal operation point, the sharpest asymmetric resonant peak, the most remarkable Fano line shape, and the highest ER (34 dB) can be obtained. Along with the increase in the bias voltage, on the one hand, the spectra can be redshifted if the arm coupled with a PCNC is biased. On the other hand, the transmission spectra can also be blueshifted if the other arm of the MZM is biased. It can be observed from Figure 8c that the FSR equals to 20 nm; then, the *V_π_L* that equals to 3.9 V·cm can be calculated using Equation (7). Thus, according to the spectra in Figure 8d, the Fano resonance effects prove the feasibility of our design. Therefore, the advantages of our design for a Fano resonance MZM such as high ER, high modulation efficiency, and high integration will be beneficial to more quantization levels, more efficient matrix multiplication, and lower power consumption for silicon photonic on-chip computing.

In order to provide a more comprehensive comparison between our design for the Fano resonance modulator and the existing designs, Table 2 presents a summary of the performance parameters of the modulators with different structures. Overall, by comparison with the existing schemes of the modulators, it is seen from Table 2 that our Fano resonance modulator design has comparatively superior performance, with the obvious advantages of the ER and the PCNC radius (that are beneficial to fabrication compatibility with a passive MPW). If non-silicon materials are heterogeneously integrated, such as thin-film lithium niobate, it may further improve the modulation speed, efficiency, and integration, as well as offer more possibilities for the suppression of third-order nonlinear distortion.

## 5. Conclusion and Perspectives

In this paper, a Fano resonance MZM based on a single arm coupled with a PCNC is designed and theoretically analyzed. By means of free-carrier dispersion effects, bias voltages are applied to our Fano resonance modulator, and the optimal driving voltage of 4.3 V is obtained. Additionally, the interference between the discrete states from one arm coupled with a PCNC and the continuous states from the other arm of the MZM generates a Fano resonance at the output port of an MMI, and 180 nm passive silicon photonic facilities are considerably applicable to the fabrication of a PCNC with identical hole radii of 90 nm. To verify the component performance, an FDTD model is utilized by numerical simulations to prove that *N_taper_*, *N_mirror_*, *A_taper_*, *a*, and gap notably affect the transmission spectra of the PCNC, obtaining the corresponding optimized parameters of 40, 5, 0.43 µm, 0.3 µm, and 0.18 µm, respectively. Moreover, the adjustable doping concentration and the length of the active region determine a modulation efficiency of 3.9 V·cm. Compared with the same structure without a PCNC, our modulator with a single arm coupled with a PCNC has a sharper Fano line shape and possesses a higher ER, as well as a higher modulation efficiency, which can introduce a speedy variation in the optical intensity from minimum to maximum. Therefore, our design for a silicon Fano resonance MZM based on a single arm coupled with a PCNC has considerable potential for applications in the fields of photonic computing, photonic convolutional neural network, optical communications, optical interconnects, optical storage, and so on in the future.

## Figures and Tables

**Table 1:** Design and performance parameters of modulator.

D_P++_	D_N++_	D_P+_	D_N+_	D_P_	D_N_
1 × 10^20^/cm^3^	1 × 10^20^/cm^3^	5 × 10^18^/cm^3^	5 × 10^18^/cm^3^	9 × 10^17^/cm^3^	7 × 10^17^/cm^3^
W_P++_	W_N++_	W_P+_	W_N+_	W_P_	W_N_
4.275 µm	4.275 µm	300 nm	300 nm	515 nm	335 nm
W_S_/W_G_	G_1_	H_elec_	*V_π_L*	Loss	C
50 µm	5 µm	2 µm	1.6 V·cm	3.3 dB	0.244 fF/µm

**Table 2:** Comparison of the performances of the modulators with different structures.

Structure	Footprint	ER [dB]	*V_π_L* [V·cm]	Radius [nm]	Fano Resonance	Refs.
MZM coupled with PCNC	W: 25 µm L: 2 mm	34	3.9	90	Yes	This work
Photonic crystal waveguide MZM	W: 200 µm L: 1.057 mm	11	7	107.5	Yes	[21]
PCNC loaded GAMS	/	< 10	/	Min: 26.63 Max: 113.13	Yes	[22]
MRR assisted MZM	W: 140 µm L: 600 µm	/	0.96	/	Yes	[23]
TFLN–based MZM	W: 500 µm L: 5 mm	4.7	1.02	/	No	[24]

## Data Availability

The data are available on request.

## Abbreviations

The following abbreviations are used in this manuscript:

PCNC	Photonic Crystal Nanobeam Cavities
MZM	Mach–Zehnder Modulator
ER	Extinction Ratio
EOM	Electro-optic Modulators
MMI	Multi-Mode Interferometer
Q-factor	Quality Factor
FDTD	Finite-Difference Time-Domain

## References

[1] Bangari V., Marquez B.A., Miller H., Tait A.N., Nahmias M.A., Lima T.F., Peng H.T., Prucnal P.R., Shastri B.J. (2019). Digital electronics and analog photonics for convolutional neural networks (DEAP-CNNs). IEEE J. Sel. Top. Quantum Electron..

[2] Wang L., Zhang Y., Wang D., Hao T., Chong Y., Gu Y., Li G., Li M., Xiao X., Zhu N. (2022). Photonic generation of multi-format radar waveforms based on an integrated silicon IQ modulator. IEEE J. Sel. Top. Quantum Electron..

[3] Hou S., Hu H., Liu Z., Xing W., Zhang J., Hao Y. (2024). High-Speed Electro-Optic Modulators Based on Thin-Film Lithium Niobate. Nanomaterials.

[4] Mao J., Uemura F., Yazdani S.A., Yin Y., Sato H., Lu G., Yokoyama S. (2024). Ultra-fast perovskite electro-optic modulator and multi-band transmission up to 300 Gbit s^−1^. Commun. Mater..

[5] Salmani M., Eshaghi A., Luan E., Saha S. (2021). Photonic computing to accelerate data processing in wireless communications. Opt. Express.

[6] Xu X.Y., Jin X.M. (2023). Integrated photonic computing beyond the von Neumann architecture. ACS Photon..

[7] Siew S.Y., Li B., Gao F., Zheng H., Zhang W., Guo P., Xie S.W., Song A., Dong B., Luo L.W. (2021). Review of silicon photonics technology and platform development. J. Light. Technol..

[8] Sia J.X.B., Li X., Wang J., Wang W., Qiao Z., Guo X., Lee C.W., Sasidharan A., Gunasagar S., Littlejohns C.G. (2022). Wafer-scale demonstration of low-loss (~0.43 dB/cm), high-bandwidth (>38 GHz), silicon photonics platform operating at the C-band. IEEE Photon. J..

[9] Li M., Wang L., Li X., Xiao X., Yu S. (2018). Silicon intensity Mach-Zehnder modulator for single lane 100 Gb/s applications. Photon. Res..

[10] Wang C., Zhang M., Chen X., Bertrand M., Shams-Ansari A., Chandrasekhar S., Winzer P., Lončar M. (2018). Integrated lithium niobate electro-optic modulators operating at CMOS-compatible voltages. Nature.

[11] Liao L., Samara-Rubio D., Morse M., Liu A., Hodge D., Rubin D., Keil U.D., Franck T. (2005). High speed silicon Mach-Zehnder modulator. Opt. Express.

[12] Xiao X., Li M., Wang L., Chen D., Yang Q., Yu S. High speed silicon photonic modulators. Proceedings of the Optical Fiber Communication Conference.

[13] Lee B.Y., Tsai C.T., Lin H.S., Kao S.C., Cheng C.H., Chiang P.Y., Lee H.C., Lin G.R. (2021). C-Band silicon waveguide modulation with 50-Gbit/s NRZ-OOK over 10-km SMF and DSF. IEEE J. Quantum Electron..

[14] Yang D.Q., Duan B., Liu X., Wang A.Q., Li X.G., Ji Y.F. (2020). Photonic crystal nanobeam cavities for nanoscale optical sensing: A review. Micromachines.

[15] Casas F.J., Pascual-Cisneros G. (2023). Mach–Zehnder Modulators for Microwave Polarization Measurement in Astronomy Applications. Sensors.

[16] Ehteshami N., Zhang W., Yao J. (2016). Optically tunable full 360° microwave photonic phase shifter using three cascaded silicon-on-insulator microring resonators. Opt. Commun..

[17] Agruzov P., Parfenov M., Tronev A., Varlamov A., Ilichev I., Usikova A., Shamrai A. (2022). Electronic System of Remote Optical Control of LiNbO_3_ Mach-Zehnder Modulator Operating Point. Electronics.

[18] Krause E.E., Malka D. (2023). Optimizations of double titanium nitride thermo-optic phase-shifter heaters using SOI technology. Sensors.

[19] Uddin M.R., Siang T.K., Munarah N., Norfauzi M., Ahmed N., Salam M.A. Quality analysis of a photonic micro-ring resonator. Proceedings of the 2016 International Conference on Computer and Communication Engineering (ICCCE).

[20] Donzella V., Sherwali A., Flueckiger J., Grist S.M., Fard S.T., Chrostowski L. (2015). Design and fabrication of SOI micro-ring resonators based on sub-wavelength grating waveguides. Opt. Express.

[21] Nguyen H.C., Yazawa N., Hashimoto S., Otsuka S., Baba T. (2013). Sub-100 µm photonic crystal Si optical modulators: Spectral, athermal, and high-speed performance. IEEE J. Sel. Top. Quantum Electron..

[22] Liu H., Liu P., Bian L., Liu C., Zhou Q. (2018). Electro-optic modulator side-coupled with a photonic crystal nanobeam loaded graphene/Al_2_O_3_ multilayer stack. Opt. Mater. Express.

[23] Chen S., Zhou G., Zhou L., Lu L., Chen J. (2020). High-linearity Fano resonance modulator using a microring-assisted Mach—Zehnder structure. J. Light. Technol..

[24] Meng X., Yuan C., Cheng X., Yuan S., Shang C., Pan A., Qu Z., Wang X., Wang J., Zhang P. (2025). Thin-Film Lithium Niobate Modulators with Ultra-High Modulation Efficiency. Laser Photon. Rev..

[25] Zhang J., Han L., Kuo B.P.P., Radic S. (2020). Broadband angled arbitrary ratio SOI MMI couplers with enhanced fabrication tolerance. J. Light. Technol..

[26] Sharma B., Kishor K., Pal A., Sharma S., Makkar R. (2021). Design and simulation of ultra-low loss triple tapered asymmetric directional coupler at 1330 nm. Microelectron. J..

[27] Liu K., Fan H., Feng S., Guo H., Li C. (2019). Three-output-channel photonic crystal splitter and switch based on nonlinear resonators and the self-collimation characteristics of a light beam. Appl. Opt..

[28] Bekele D., Yu Y., Yvind K., Mork J. (2019). In-Plane Photonic Crystal Devices Using Fano Resonances. Laser Photon. Rev..

[29] Tzuang L.D., Soltani M., Lee Y.H.D., Lipson M. (2014). High RF carrier frequency modulation in silicon resonators by coupling adjacent free-spectral-range modes. Opt. Lett..

[30] Soref R., Bennett B. (1987). Electrooptical effects in silicon. IEEE J. Quantum Electron..

[31] Reed G.T., Mashanovich G., Gardes F.Y., Thomson D.J. (2010). Silicon optical modulators. Nat. Photon..

[32] Sun F., Li Z., Tang B., Li B., Zhang P., Liu R., Yang G., Huang K., Han Z., Luo J. (2023). Scalable high Q-factor Fano resonance from air-mode photonic crystal nanobeam cavity. Nanophotonics.

